# Personal Quality of Life as an Outcome Measure of Antipsychotic Drug Management of Problem Behaviours in Adults with Intellectual Developmental Disorders with or Without Autism Spectrum Disorder

**DOI:** 10.3390/brainsci15030316

**Published:** 2025-03-18

**Authors:** Marco O. Bertelli, Annamaria Bianco, Micaela Piva Merli, Maria Stella Vescio, Michele Rossi, Elisabetta F. Buonaguro

**Affiliations:** 1 CREA (Research and Clinical Centre), San Sebastiano Foundation, Misericordia di Firenze, 50100 Florence, Italy; abianco@crea-sansebastiano.org (A.B.); mpiva@crea-sansebastiano.org (M.P.M.); mvescio@crea-sansebastiano.org (M.S.V.); mrossi@crea-sansebastiano.org (M.R.); 2 DSNP (Department of Neuro Psychiatric Sciences), University of Florence, 50100 Florence, Italy; 3 Section of Psychiatry, Department of Neuroscience, Reproductive Science and Odontostomatology, University of Naples Federico II, 80100 Naples, Italy; elisabetta.buonaguro@unina.it

**Keywords:** intellectual disability, autism, antipsychotics, problem behaviours, quality of life

## Abstract

**Background/Objectives:** First-generation antipsychotics (FGAs) and second-generation antipsychotics (SGAs) are frequently prescribed for the management of problem behaviours (PBs) in people with intellectual developmental disorders (IDDs) with or without autism spectrum disorder (ASD). However, the different effectiveness of these two groups of drugs has not been adequately investigated, especially in terms of person-centred outcomes, such as quality of life (QoL). The aim of the present observational study was to compare the personal QoL of two groups of people with IDDs with and without ASD, attending the same residential facility, but receiving FGAs and SGAs, respectively, for the management of PBs. **Methods:** Twenty-two people with IDDs (ten with ASD) receiving FGAs and twenty-three (eleven with ASD) receiving SGAs for the management of PBs were consecutively recruited. The participants underwent a complex clinical and instrumental evaluation, including the systematic psychopathological assessment for persons with intellectual and developmental disabilities—general screening (SPAIDD-G), the Wing’s handicaps, behaviours, and skills schedule (HBS), the DOTES, and the BASIQ (QoL Assessment tool battery). Follow-up evaluations were carried out after 6, 12, and 18 months. **Results:** The use of antipsychotics was withdrawn only in 16 cases, of which only 4 were for significant improvement. Treatment with FGAs was associated with more frequent discontinuation, a higher incidence of side effects, and a trend toward a lower efficacy on PBs than treatment with SGAs. FGAs also improved generic QoL significantly less than SGAs. **Conclusions:** The present pilot study is the first to compare FGAs and SGAs with respect to the generic QoL in people with IDDs and PBs. SGAs resulted to have a significantly greater positive impact on QoL than FGAs, despite demonstrating similar efficacy in treating PBs.

## 1. Introduction

Problem behaviours (PBs) represent frequent issues in clinical practice of healthcare workers caring for people with intellectual developmental disorders (PwIDDs) [1]. The term ‘intellectual developmental disorders’, also addressed as ‘intellectual disability’ [2] or ‘disorders of intellectual development’ [3], refers to a meta-syndromic group of conditions sharing cognitive and adaptive issues with onset in early childhood. PBs are commonly defined as ‘socially unacceptable behaviours that cause distress, harm, or disadvantage to the persons themselves or to other people’ [4]. They usually require some intervention and can typically include a range of manifestations such as self-injury, aggression, stereotypes, pica, and rumination. The presence of PBs can have serious disadvantages such as interference with quality of life (QoL) and social participation of PwIDDs [5].

It is often difficult to determine whether PBs are supported by genetic causes (behavioural phenotypes), physical pain and/or other physical problems, drug-related side effects, psychological, environmental, socio-relational factors, or even a combination of some of these aspects [6]. Nevertheless, some PBs can represent symptoms, or groups of symptoms, of specific psychiatric disorders (PDs), taking the name of ‘behavioural equivalents’ (BEs) [7]. These can be characteristic for onset, development, maintenance, and extinction, especially with respect to other concurrent possible symptoms of a PDs. Therefore, an assessment of psychiatric co-occurrences can have a relevant impact on the understanding of the manifestations of PBs in PwIDDs.

While pharmacological interventions for PBs in PwIDDs should be reserved as a last resort, used for the shortest duration possible, and administered at the lowest effective dose [8], their use is often excessive, prolonged, and at high dosages. The prescription of antipsychotics (APs), in particular, is a highly debated topic in mental health, as they are reportedly the most frequently prescribed medication for this population [9]. Specifically, APs are used to control PBs in 19–58% of PwIDDs [9,10,11,12].

Among the typical antipsychotics (APs), also referred to as first-generation APs (FGAs), the most frequently prescribed are haloperidol, thioridazine, and chlorpromazine. Studies on the safety of these compounds show the onset of severe motor and cardiac side effects in PwIDDs (sedation, cognitive performance reduction, dystonia and extrapyramidal signs, QTc prolongation, tardive dyskinesia, or neuroleptic malignant syndrome) [13].

Notably, practical guidelines were published in 2009 for the use of second-generation APs (SGAs) in adults with IDDs [14]. Compared to FGAs, SGAs continue to share dopamine D2 receptor affinity at different degrees but also present an overall different receptor binding profile, with the result of having a putative lower negative impact on systems that are suggested to be more vulnerable in PwIDDs, such as neuro-motor and cognitive ones [15,16].

The exploration of the efficacy of SGAs in comparison to FGAs on PBs in PwIDDs could have important repercussions on daily practice, in consideration of the different impacts that the two pharmacologic classes potentially have on new person-centred outcome measures, such as QoL. Indeed, QoL has gradually gained importance and interest and has become a broadly used concept in the care for PwIDDs [10]. Interestingly, several studies have found that QoL is strongly associated with health status (mental or physical wellbeing) and the presence of PBs [17,18]. QoL is a polysemic expression referring to a multidimensional concept and it includes various areas of human life like health, physical functioning, psychological and spiritual well-being, social roles, economic stability, and family functioning. An important distinction must be drawn between personal and shared (common to every person) aspects of QoL [19]. Another relevant differentiation has to be made between health-related QoL (HR-QoL) and generic QoL (G-QoL) or, as it was named by Brown and Brown [20] whole-person QoL. HR-QoL, by its very nature, is closely associated with shared aspects of QoL that overlap with symptomatic and dysfunctional aspects, aiming to repurpose a morphological and functional model of normality [19]. Generic personal QoL models assess the subjective perception of quality in the context of the individual’s own body, environment, and culture, with respect to all of the areas of his/her life that constitute importance and value (independent of the presence of pathologies). Therefore, in terms of QoL, the value of an outcome measure related to psychopharmacological treatment regimens could also be related to a patient’s perception of treatment adequacy and utility towards his own life, beyond its efficacy on symptoms and tolerability. To our knowledge, no study has specifically addressed the measurement of the effects of the use of APs for the management of PBs on personal QoL in PwIDDs. Very few studies have considered its relationship with HR-QoL, intended exclusively as the presence or pervasiveness of symptoms and disabilities, with reference to standard life activities [10,21].

Starting from these premises, the present study aimed to verify the hypothesis of a different impact of the use of FGAs vs. SGAs on personal QoL for the management of PBs in a population of PwIDDs living in residential facilities.

## 2. Materials and Methods

### 2.1. Study Design

The study was a two-arm, parallel group pragmatic trial of FGA and SGA-treated subjects, with a blind assessment of the outcomes at three time points (6, 12, and 18 months) after the beginning of the assigned treatment with APs.

### 2.2. Sample

Participants were consecutively recruited among attendants of a rehabilitative and residential centre in Tuscany, Italy, after their psychiatrists and their multidisciplinary care teams had decided to prescribe APs for the management of PBs. The medications used were chosen by the treating clinician on the basis of symptomatologic characterization and pharmacological anamnesis. Inclusion criteria also comprised documentation of the diagnosis of an IDD, in accordance with DSM-5 criteria [2]. Twenty-two subjects were prescribed with an FGA drug and twenty-three with an SGA.

### 2.3. Procedure and Assessments

Participants were evaluated during a total period of 18 months. At baseline, all participants were clinically evaluated and diagnosed for co-occurrence of psychopathological conditions, in accordance with DSM-5 and DM-ID criteria [22]. The diagnoses were conducted by two experienced psychiatrists, who had a good knowledge of the basic behaviour and functioning level of each participant. All diagnoses were made with the support of the administration of the systematic psychopathological assessment for persons with intellectual and developmental disabilities—general screening (SPAIDD-G) [23].

Expert assessors, who were blinded to the medications being used, evaluated the patients after 6, 12, and 18 months with the above-listed tools. For all cases, APs were started after previous non-pharmacological or pharmacological interventions had failed.

Clinical laboratory tests, including complete blood count, fasting glucose, prolactin, lipid profile, kidney and liver function indexes, and weight measurements, were performed at baseline and after 6, 12, and 18 months. The presence of cardiac abnormalities was assessed by 12-lead electrocardiogram (ECG) during all treatment phases and at every dosage variation. Specific monitoring was performed for the participants treated with clozapine according to the current guidelines.

### 2.4. Instruments

During the baseline clinical evaluation, every participant was assessed using the SPAIDD-G [23]. This tool represents the general version of the SPAIDD diagnostic system, which is based on the detection of psychiatric symptoms starting from the observation of attitudes and behaviours, which is the only survey method applicable to all levels of IDD severity [23].

At the follow-up assessments, all participants were evaluated using Wing’s handicaps, behaviours, and skills schedule (HBS), the dosage record and treatment emergent symptoms scale (DOTES), and the BASIQ (batteria di strumenti per l’indagine della qualità di vita) QoL assessment tool [24].

#### 2.4.1. Wing’s Handicap, Behaviours, and Skills Schedule (HBS)

The HBS was used to assess the severity of PBs. This schedule is a clinical interview designed to explore details concerning many aspects of development, including social interaction, non-verbal and verbal communication, imaginative play; and abnormalities of behaviour, including repetitive routines. Higher scores indicate a higher presence and pervasiveness of PBs. The schedule has been widely used with adults with IDDs on patterns of abnormality of psychological function [25].

#### 2.4.2. Dosage Record and Treatment Emergent Symptoms Scale (DOTES)

DOTES was used to assess side effects of APs. DOTES is a comprehensive scale that measures the presence and intensity of adverse effects induced by psychotropic medications [26].

#### 2.4.3. BASIQ (BAtteria di Strumenti per l’Indagine Della Qualità di Vita) QoL Assessment Tool

Personal QoL was assessed using the proxy version of BASIQ. BASIQ is the Italian adaptation of the QoL instrument package that was released by the QoL Research Unit at the Department of Occupational Therapy of the University of Toronto, Toronto, ON, Canada [27]. The package is based on an interpretative model of QoL that integrates qualitative and quantitative as well as subjective and objective data, but the core QoL measure is the perception of satisfaction weighted by ratings of importance, as expressed by a combination of a persons’ self-expressions and systematic interpretation of their behavioural repertories by their proxies.

The nine areas of life tackled by the questionnaire are gathered into the three macro-areas of Being, Belonging, and Becoming. ‘Being’, which defines the principal characteristics of a person, can be divided into Physical, Psychological, and Spiritual. ‘Belonging’ describes the relationship between a person and the surrounding environment and is divided into Physical, Social, and Community. ‘Becoming’ describes everything a person does in life and what one wants to become. It is divided into Practical, Leisure, and Growth.

The nine areas represent areas of life that various international research paths have showed to be of qualitative value to all people around the world [19].

Before the administration of BASIQ and all of the other above-mentioned scales, a high inter-rater reliability (>0.75) was ascertained through a special session in which different professionals independently attributed scores to the same case, after having been provided information by the referring psychiatrist.

### 2.5. Data Elaboration and Statistical Analysis

Antipsychotic doses were converted into defined daily dose (DDD) in accordance with the definition of the World Health Organization [28]. This method was chosen since: (1) equivalent dosing is important to guarantee comparisons of drugs; (2) DDD is frequently used in psychopharmacology research since it is available for most APs; (3) all other approaches to define the equivalent dosing of APs are currently only available for a selection of them; (4) DDD is an internationally accepted measure.

The AP doses equivalent to oral olanzapine were calculated according to the DDD method, and the mean of the DDD of APs for each group was compared at the different time points using Student’s *t* test.

Statistical comparison between the two groups on the background characteristics and HBS and QoL scores was conducted using Student’s *t* test for continuous variables, with Welch and Bonferroni corrections. Pearson’s r correlation (checked by Spearman’s ρ) was conducted for the HBS and QoL mean scores at the different time points.

Statistical analyses were conducted using SPSS version 16.0 for Windows.

### 2.6. Ethics

The study was conducted in accordance with the Declaration of Helsinki. All participants provided formal written informed consent before entering into the study. For those who were unable to do it by themselves, the consent was given by their guardians. Privacy was assured by not recording names or any other identifying information on the written material. Data were stored in a secure location. The study was approved by the Tuscan Scientific Association for Rehabilitation Service Providers on 27 November 2020, with the code ‘APP_01–QuAC’, also in reference to ethical issues.

## 3. Results

### 3.1. Background Characteristics

Baseline characteristics of the sample were collected at the beginning of the study for the two different treatment groups. The group assigned to FGAs comprised twenty-two adults (mean age of 38.7 ± 8.3 years), of which twenty were male and two were female, with a mean IDD severity level of 1.84 ± 0.7 (range of 1–4, from mild to profound). Co-presence of ASD was documented in ten participants. The group assigned to SGAs included 23 adults (mean age of 39.2 ± 9.4 years), of which twenty were male and three were female, with a mean IDD severity level of 1.78 ± 0.9. Co-presence of ASD was documented in eleven participants.

### 3.2. Psychopathological Co-Occurrences

The diagnoses of the participants, which were obtained using SPAIDD-G screening and clinically confirmed in accordance with the DM-ID and DSM-5 criteria, were schizophrenia (18.18% for the FGA group and 21.74% for the SGA group), impulse-control disorder (22.73% for the FGA group and 21.74% for the SGA group), obsessive–compulsive disorder (4.55% for the FGA group and 4.35% for the SGA group), bipolar disorder type I (18.18% for the FGA group and 21.74% for the SGA group), and IDDs without psychopathological co-occurrence in only two cases (4.55% for the FGA group and 4.55% for the SGA group). General co-occurrence of psychopathology was high (22.73% for the FGA group and 21.74% for the SGA group). These results are summarised in Table 1.

### 3.3. Compounds and Dosages

Within the FGA group, eleven subjects (50%) received haloperidol, four (18.18%) received chlorpromazine, three (13.64%) received levomepromazine, two (9.9%) received promazine, and two (9.9%) received perphenazine. In the SGA group, ten (43.48%) received clozapine, six (26.09%) received olanzapine, five (21.74%) received quetiapine, and two (8.7%) received risperidone.

The different compounds in the two subsamples and their mean dosage by time (T1, T2, and T3) are reported in Table 2.

**Figure 1:**
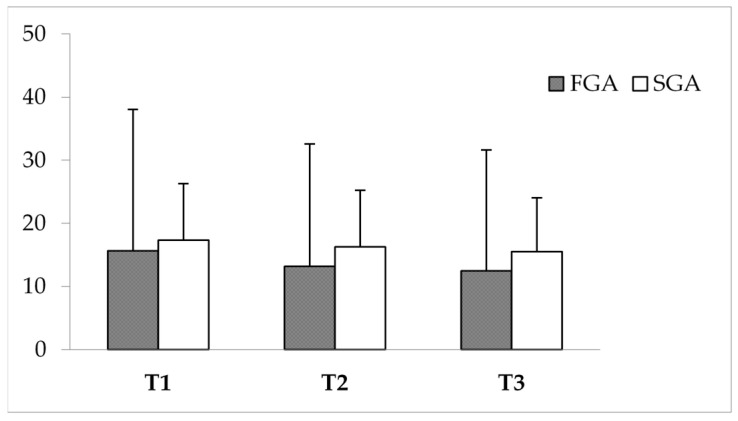
Mean dosages of FGAs and SGAs at T1, T2, and T3. Dosages are expressed in DDD olanzapine equivalents. FGAs, first-generation antipsychotics (grey); SGAs, second-generation antipsychotics (white).

There was no statistically significant difference in terms of DDD between the FGA and SGA-treated groups at the different time points of the study (*p* > 0.05; see Figure 1).

### 3.4. AP Discontinuation

Successful discontinuation of antipsychotic medication, based on the clinical judgment that PBs could be managed solely through non-pharmacological interventions, was achieved in only four participants. All four discontinuations occurred at T3, with one participant in the FGA group and three in the SGA group.

### 3.5. Impact of APs on PBs

HBS scores collected at T0, T1, T2, and T3 were different in the two groups. At T0, HBS scores were 18.24 ± 4.2 for the FGA group and 18.17 ± 3.9 for the SGA group; at T1, 15.34 ± 3.6 for the FGA group and 16.02 ± 3.8 for the SGA group; at T2, 13.6 ± 2.9 for the FGA group and 12.17 ± 2.7 for the SGA group; and at T3, 11.2 ± 2.3 for the FGA group and 7.8 ± 1.8 DS for the SGA group.

**Figure 2:**
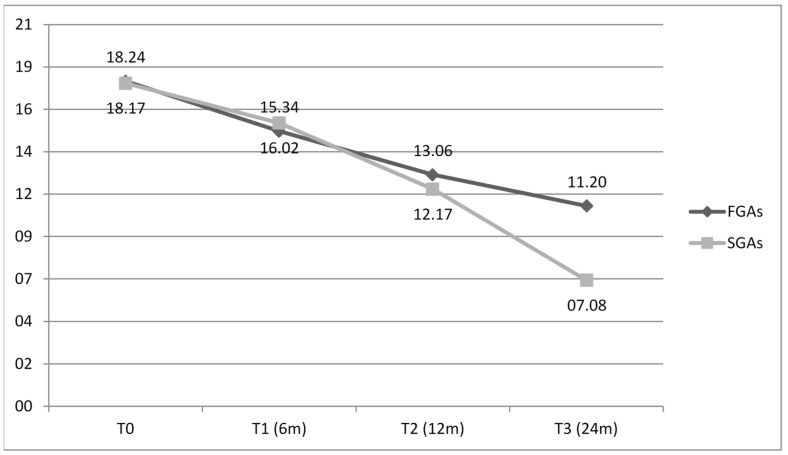
HBS scores by time. FGAs, first-generation antipsychotics (black); SGAs, second-generation antipsychotics (grey). HBS score does not show statistically significant difference between the two AP groups (*p* ≤ 0.05).

HBS scores did not show any statistically significant differences between the two treated groups (*p* ≤ 0.05). Nevertheless, PBs measured by severity and frequency showed a greater decrease in the SGA group compared to the FGA group (see Figure 2).

### 3.6. Impact of APs on QoL

SGAs led to a significantly greater improvement in QoL compared to FGAs, despite showing comparable efficacy in treating PBs. Specifically, significant differences favouring SGAs were observed in the ‘Being’ (Psychological) and ‘Becoming’ (Practical, Leisure Time, and Personal Growth) domains of QoL.

**Figure 3:**
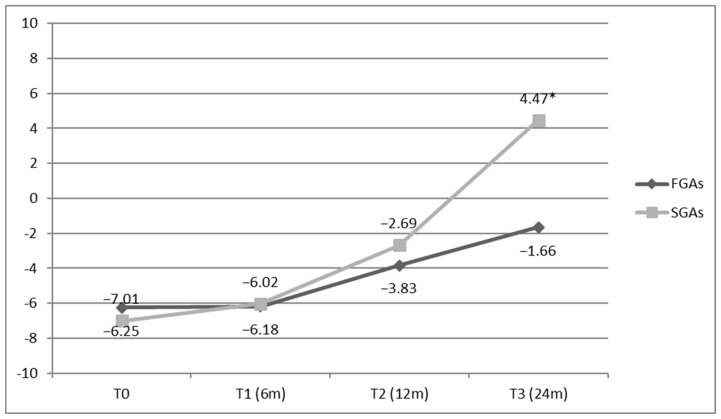
Overall BASIQ (QoL) scores by time. FGAs, first-generation antipsychotics (black); SGAs, second-generation antipsychotics (grey). The asterisk (*) adjacent to the SGAs value at T3 signifies a statistically significant difference between the FGA and SGA groups (*p* < 0.001).

Overall BASIQ scores collected at T0 were −6.25 ± 2.6 for the FGA group and −7.01 ± 2.9 for the SGA group; at T1, −6.18 ± 2.4 for the FGA group and −6.02 ± 2.2 for the SGA group; at T2, −3.83 ± 1.7 for the FGA group and −2.69 ± 1.3 for the SGA group; and at T3, −1.66 ± 0.6 and 4.47 ± 1.4 for the SGA group. At T3, the score difference was statistically significant (*p* < 0.001). These results are illustrated in Figure 3.

**Figure 4:**
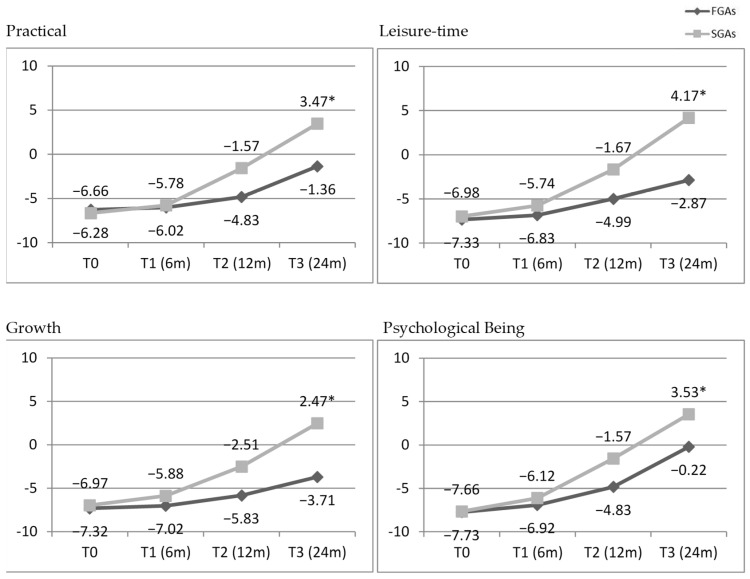
BASIQ areas with significant endpoint score differences (FGA vs. SGA scores by time. FGAs, first-generation antipsychotics (black); SGAs, second-generation antipsychotics (grey). The asterisk (*) adjacent to the SGAs value at T3 signifies a statistically significant difference between the FGA and SGA groups (*p* < 0.001).

In reference to specific QoL areas, statistically significant differences in the BASIQ scores of FGA and SGA-treated groups were found in the macro areas of Being and Becoming, namely in the subareas of and Being—Psychological, Becoming—Practical, Becoming—Leisure time, and Becoming—Growth. Score details are shown in Figure 4.

### 3.7. Correlation Between PBs and QoL

**Figure 5:**
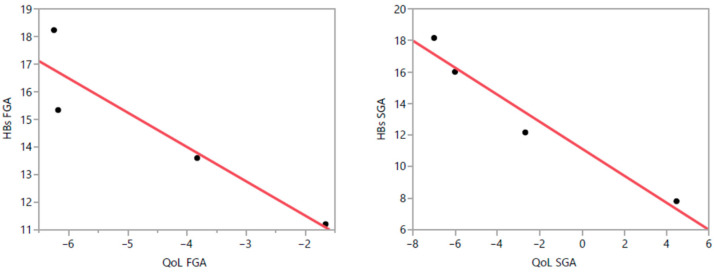
HBS and BASIQ Pearson’s correlation analyses in FGAs and SGA groups of treatment. A negative relationship was found in both groups, but it was statistically significant only in the SGA group, indicating that higher HBS scores (more severe PBs) were significantly associated with lower BASIQ scores (worse QoL) in that group. Dots refer to the combination of QoL and HBS scores at T0, T1, T2, and T3 while the red line represents the line of best fit obtained through linear regression analysis.

The correlation analyses indicated that HBS mean scores at the different time points were negatively correlated with overall BASIQ mean scores in both the FGA (r = −0.921; *p* = 0.079) and SGA groups (r = −0.976, *p* = 0.024), but only in the latter group was the correlation statistically significant. This difference was also found for BASIQ sub-areas (as follows). Becoming—Practical: FGAs r = −0.891, *p* = 0.109; SGAs r = −0.992, *p* = 0.008. Becoming—Leisure Time: FGAs r = −0.950, *p* = 0.05; SGAs r = −0.992, *p* = 0.008. Becoming—Growth: FGAs r = −0.928, *p* = 0.072; SGAs r = −0.992, *p* = 0.008. Being—Psychological: FGAs r = −0.928, *p* = 0.072; SGAs r = −0.997, *p* = 0.003. Graphics of the correlation analyses are reported in Figure 5.

### 3.8. Treatment Interruption and Side Effects

Of the forty-five participants originally recruited into the study, twelve discontinued during the full 18-month trial period (ten patients treated with FGAs and two patients treated with SGAs).

According to the DOTES assessment, Table 3 shows the main cause of discontinuation from adverse events and the frequency of side effects.

## 4. Discussion

### 4.1. Over-Prescription and Concerns of AP Use for PBs in IDD

The successful discontinuation of APs—based on the clinical judgment that PBs could be managed solely through non-pharmacological interventions—was only achieved in four participants. Unfortunately, this finding is not uncommon in the current management of PBs in many rehabilitative, residential, and clinical services across Europe and worldwide [29].

In fact, the literature indicates that psychotropic medications are commonly prescribed to individuals with IDDs, with prescription rates ranging from 32% to 85%, and an average of 50% to 63%. APs are the most frequently used psychotropic medication, with 21% of adults with IDDs receiving them. This is significantly higher than the general population, where AP use is less than 1%. In many cases, APs are prescribed without a diagnosed mental illness (36% to 71%), primarily for the management of PBs [30].

The widespread off-label use of APs for individuals with IDDs raises significant public health concerns. It is estimated that 35,000 adults with IDDs in England may be receiving unnecessary psychotropic medication each year, and long-term AP use poses risks to QoL due to potential adverse effects [30]. This prompted NHS England to launch the STOMP (stopping over-medication of people with an intellectual disability, autism, or both) campaign in 2016 [31].

On the other hand, there are clinical studies, multiple meta-analyses, and systematic reviews reporting some effectiveness of APs on PBs [32,33,34,35,36]. In our study, both FGAs and SGAs appeared effective in reducing PBs. Patients initiating FGA treatment presented with similar levels of PBs compared to those starting SGAs. While not statistically significant, our results suggest a trend towards greater PB reduction with SGAs, potentially indicating superior efficacy independent of dosage. This is supported by the observation that there were no significant differences in the mean DDD between FGAs and SGAs at any of the three time points. However, larger studies are needed to confirm these findings.

### 4.2. AP Side Effects

Individuals with IDDs and/or ASD are particularly vulnerable to AP side effects, which can substantially affect individual functioning, including cognitive performance and QoL. Movement-related side effects are especially prevalent and may worsen PBs. Our study reflected this, revealing numerous side effects (especially movement-related side effects) primarily within the FGA group, where they were the leading cause of treatment discontinuation. Metabolic effects were observed mainly in the SGA group. Overall, SGAs demonstrated better tolerability than FGAs, resulting in only two discontinuations (both due to weight gain and dysmetabolism) compared to ten discontinuations in the FGA group.

It is important to note that in people with an IDD, the use of APs is associated with an unmodifiable risk of tardive dyskinesia, higher than in the general population. Modifiable comorbidity and treatment-related risk factors include treatment with FGAs versus SGAs, higher cumulative and current antipsychotic dose or plasma levels, early parkinsonian side effects, and concurrent anticholinergic medication [37].

### 4.3. PBs and Psychopathology

A significant number of individuals with IDDs and/or ASD receive medication, often APs, without the benefit of a thorough psychiatric evaluation and diagnosis. Best practice dictates that medication, particularly APs, should be prescribed only with clearly defined objectives and after robust assessment and adherence to established clinical procedures. This approach should be grounded in principles of interdisciplinary collaboration, precision, personalised care, and active patient participation [38]. Despite using IDD-specific screening and diagnostic tools, we found that roughly 38% of FGA users and 35% of SGA users in our sample lacked a documented diagnosis justifying AP treatment.

The interplay between PBs and psychopathological co-occurrence in individuals with IDDs remains a complex and poorly understood area. While the precise nature of this relationship requires further investigation, it is plausible that mental health conditions contribute to some PBs, potentially manifesting as atypical presentations of psychiatric disorders or emerging secondary to these disorders.

In the present study, we found that co-occurrence of psychiatric conditions was high, with relevant percentages of impulse control disorder, bipolar disorder type I, and schizophrenia in both groups. Consequently, it could be hypothesised that at least a cluster of PBs recorded in the study population could represent the manifestation of co-occurrent psychopathology.

### 4.4. AP Discontinuation

Both national and international guidelines advocate for regular reviews of AP medication, emphasizing reduction, discontinuation, or minimizing the duration of use. One practical approach to combatting overmedication involves tapering AP dosages. Research in the UK and the Netherlands demonstrates the feasibility of completely discontinuing APs in 25% to 46.5% of long-term users, with an additional 11% to 19% achieving dose reductions exceeding 50% [9,39,40,41].

Several factors influence withdrawal success. Research has identified associations between positive outcomes and lower initial antipsychotic doses, minimal psychopathology, and the absence of aggression, stereotypy, and hyperactivity. Conversely, factors like female gender, lower baseline PBs, and lower baseline AP dosage are favourable, while severe PBs and autonomic and extrapyramidal symptoms pose challenges. Comorbid ASD, higher AP doses, greater severity of PBs, higher akathisia scores, and worsening health during withdrawal are also linked to lower rates of complete discontinuation and can influence reinstatement rates [42].

While AP discontinuation often improves PBs and QoL, it can also trigger withdrawal symptoms, potentially manifesting as PBs. It is crucial, however, to differentiate withdrawal-related behavioural changes from other potential causes of deterioration. A comprehensive behavioural assessment is essential, as behavioural changes may be unrelated to the withdrawal process itself.

### 4.5. Impact of APs on QoL

Regarding QoL, SGAs impacted personal QoL significantly better than FGAs. This difference likely stems from a greater ability of SGAs to help individuals achieve a better balance between the importance they place on various life areas and their actual satisfaction in those areas. For instance, SGAs may facilitate greater satisfaction with preferred leisure activities compared to FGAs, which could be explained in turn by a less detrimental impact on an individual’s motivation and ability to engage in such activities. The varying effects of FGAs and SGAs on QoL could relate to their differing receptor binding affinities, influences on neurotransmitter activity, and overall side effect profiles. However, although to a significantly lesser extent, FGAs were also shown to improve QoL across time in our study participants. This improvement could be a result of the positive impact of APs on symptoms of co-occurrent psychopathology that influence overall QoL. However, it is also possible that factors unrelated to the specific antipsychotic used, such as behavioural therapy, environmental improvements, or other non-pharmacological interventions, are playing a role.

Direct comparison of our findings with the existing literature is challenging, as personal QoL remains an underutilised outcome measure in individuals with IDDs. QoL assessments in this population often rely on proxy reports (e.g., from parents or caregivers) focusing on health-related QoL (HR-QoL), which reflects only perceived changes in symptoms and/or disabilities. To our knowledge, only one prior study has attempted to measure QoL in individuals with IDDs and PBs, revealing a strong link between adverse events from psychotropic medications and diminished QoL [43]. However, that study employed a considerably less comprehensive QoL assessment tool than the one used in our research, notably lacking an evaluation of the personal dimensions within each life area, such as the importance attributed to (and satisfaction perceived within) those areas.

Despite limitations such as a relatively small sample size and the inclusion of individuals with moderate to severe IDDs, our study, to the best of our knowledge, is the first to utilise personal QoL measures in individuals with IDDs to compare the effects of FGAs and SGAs in managing PBs.

Given that individuals with IDDs tend to experience a lower QoL compared to the general population [19], and considering their increased vulnerability to the adverse effects of APs, research in this area warrants careful attention. This research should particularly focus on comprehensive QoL models that incorporate personal perspectives.

## 5. Conclusions

In our sample of PwIDDs living in residential facilities, SGAs had a significantly greater positive impact on QoL than FGAs, despite demonstrating similar efficacy in treating PBs. Statistically significant differences between FGAs and SGAs emerged in the QoL macro areas of Being and Becoming, specifically in the subareas of Being—Psychological, Becoming—Practical, Becoming—Leisure Time, and Becoming—Growth.

Our study has revealed the potential to identify substantial differences in QoL outcomes based on the specific classes of medication used. These findings could significantly impact daily psychiatric practice across clinical, rehabilitative, and residential settings.

However, due to the novelty and considerable implications of our results, further research with more controlled and methodologically rigorous studies is needed to confirm these findings.

## Figures and Tables

**Table 1:** Psychopathological co-occurrences, including comorbidity.

Diagnosis	Baseline (T0) N (%)	FGA Group (n = 22) N (%)	SGA Group (n = 23) N (%)
Only IDD	2 (4.5)	1 (4.6)	1 (4.4)
Schizophrenia	9 (20.0)	4 (18.1)	5 (21.7)
ICD	10 (22.2)	5 (22.7)	5 (21.7)
OCD	2 (4.5)	1 (4.6)	1 (4.4)
BD I	9 (20.0)	4 (18.1)	5 (21.7)
Schizotypical PD	1 (2.2)	1 (4.6)	-
Narcissistic PD	1 (2.2)	-	1 (4.4)
Antisocial PD	1 (2.2)	1 (4.6)	-
Comorbidity	10 (22.2)	5 (22.7)	5 (21.7)
			
Schizophrenia + SRD	3 (30.0)	2 (40.0)	1 (20.0)
BD I + SRD	1 (10.0)	-	1 (20.0)
BD I + Cluster A PD	2 (20.0)	2 (40.0)	-
BD I + Cluster B PD	3 (30.0)	1 (20.0)	2 (40.0)
BD I + Cluster C PD	1 (10.0)	-	1 (20.0)

Legend: IDD, intellectual developmental disorders; ICD, impulse control disorder; OCD, obsessive-compulsive disorder; BD I, bipolar disorder type I; PD, personality disorder; SRD, substance-related disorders).

**Table 2:** Antipsychotic treatment, % by sample and dosage by time.

Compound	N (%)	Mean Dosage mg/day		
		T1	T2	T3
**FGAs**				
Haloperidol	11 (24.4)	44.5	38.2	37.3
Chlorpromazine	4 (8.9)	200.0	162.5	137.5
Levomepromazine	3 (6.7)	91.7	91.7	83.3
Promazine	2 (4.4)	210.0	195.0	180.0
Perphenazine	2 (4.4)	16.0	8.0	7.0
				
**SGAs**				
Risperidone	2 (4.4)	6.5	5.5	5.0
Clozapine	10 (22.2)	235.0	220.5	212.5
Olanzapine	6 (13.3)	20.0	19.17	19.17
Quetiapine	5 (11.1)	1140.0	1100.0	1030.0

Legend: FGAs, first generation antipsychotics; SGAs, second generation antispychotics.

**Table 3:** Drop-outs for adverse events and frequency of side-effects by treatment.

	FGAs (n = 22)	SGAs (n = 23)
**Drop-outs for adverse events**	**N (%)**	**N (%)**
Extrapyramidal side effects	5 (22.7)	-
Electrocardiogram abnomalities	3 (13.6)	-
Abnormal laboratory test	2 (9)	1 (4.3)
Weight gain	-	1 (4.3)
**Side effects**	**N (%)**	**N (%)**
Extrapyramidal side effects	6 (27.7)	1 (4.3)
Hyperglicemia	1 (4.5)	3 (13)
Abnormalities lipid profile	1 (4.5)	5 (21.7)
Hyperprolactinemia	7 (31.8)	-
Weight gain	4 (18.1)	5 (21.7)
Sedation	4 (18.1)	3 (13)

Legend: FGAs, first generation antipsychotics; SGAs, second generation antipsychotics.

## Data Availability

Data are unavailable due to privacy or ethical restrictions.

## Abbreviations

FGAs	First-generation antipsychotics
SGAs	Second-generation antipsychotics
PBs	Problem behaviours
IDDs	Intellectual developmental disorders
ASD	Autism spectrum disorders
QoL	Quality of life
HBS	Handicaps, behaviours, and skills schedule
PwIDDs	People with intellectual developmental disorders
PDs	Psychiatric disorders
BEs	Behavioural equivalents
APs	Antipsychotics
HR-QoL	Health-related quality of life
G-QoL	Generic quality of life
SPAIDD-G	Systematic psychopathological assessment for persons with intellectual and developmental disabilities
ECG	Electrocardiogram
DOTES	Dosage record and treatment of emergent symptoms
BASIQ	Batteria di strumenti per l’indagine della qualità di vita (QoL assessment tool)
DDD	Defined daily dose
